# Endocrine Disrupting Chemicals Research Program of the U.S. Environmental Protection Agency: Summary of a Peer-Review Report

**DOI:** 10.1289/ehp.8875

**Published:** 2006-03-15

**Authors:** Anna K. Harding, George P. Daston, Glen R. Boyd, George W. Lucier, Stephen H. Safe, Juarine Stewart, Donald E. Tillitt, Glen Van Der Kraak

**Affiliations:** 1 Department of Public Health, Oregon State University, Corvallis, Oregon, USA; 2 Miami Valley Laboratories, The Procter & Gamble Company, Cincinnati, Ohio, USA; 3 HDR Engineering, Inc., Bellevue, Washington, USA; 4 Pittsboro, North Carolina, USA; 5 Department of Veterinary Physiology and Pharmacology, Texas A&M University, College Station, Texas, USA; 6 School of Computer, Mathematical and Natural Sciences, Morgan State University, Baltimore, Maryland, USA; 7 U.S. Geological Survey, Columbia Environmental Research Center, Biochemistry and Physiology Branch, Columbia, Missouri, USA; 8 Department of Integrative Biology, University of Guelph, Guelph, Ontario, Canada

**Keywords:** ecologic effects, endocrine-disrupting chemicals, endocrine disruptors, hormonal activity, human health, risk assessment, risk management, screening and testing

## Abstract

At the request of the U.S. Environmental Protection Agency (EPA) Office of Research and Development, a subcommittee of the Board of Scientific Counselors Executive Committee conducted an independent and open peer review of the Endocrine Disrupting Chemicals Research Program (EDC Research Program) of the U.S. EPA. The subcommittee was charged with reviewing the design, relevance, progress, scientific leadership, and resources of the program. The subcommittee found that the long-term goals and science questions in the EDC Program are appropriate and represent an understandable and solid framework for setting research priorities, representing a combination of problem-driven and core research. Long-term goal (LTG) 1, dealing with the underlying science surrounding endocrine disruptors, provides a solid scientific foundation for conducting risk assessments and making risk management decisions. LTG 2, dealing with defining the extent of the impact of endocrine-disrupting chemicals (EDCs), has shown greater progress on ecologic effects of EDCs compared with that on human health effects. LTG 3, which involves support of the Endocrine Disruptor Screening and Testing Program of the U.S. EPA, has two mammalian tests already through a validation program and soon available for use. Despite good progress, we recommend that the U.S. EPA *a*) strengthen their expertise in wildlife toxicology, *b*) expedite validation of the Endocrine Disruptors Screening and Testing Advisory Committee tests, *c*) continue dependable funding for the EDC Research Program, *d*) take a leadership role in the application of “omics” technologies to address many of the science questions critical for evaluating environmental and human health effects of EDCs, and *e*) continue to sponsor multidisciplinary intramural research and interagency collaborations.

At the request of the U.S. Environmental Protection Agency (EPA) Office of Research and Development (ORD), the Board of Scientific Counselors (BOSC) Executive Committee (U.S. EPA 2005a) organized and conducted an independent and open peer review of the Endocrine Disrupting Chemicals Research Program (EDC Research Program) of the U.S. EPA. In May 2004 the BOSC formed a subcommittee to conduct the review, including individuals from academia, industry, private consulting, and other agencies. The subcommittee members (co-authors of this article) have expertise specific to endocrine-disrupting chemicals (EDCs) and related areas, with research that has considerable overlap with the U.S. EPA-sponsored EDC Research Program. Three members (A. Harding, G. Daston, and J. Stewart) were also members of the BOSC Executive Committee.

The subcommittee was charged with reviewing the design, relevance, progress, scientific leadership, and resources of the program (Appendix 1) and providing a report to the BOSC Executive Committee. Our purpose was to review the progress of the U.S. EPA in establishing and managing an effective cross-disciplinary and cross-functional program in endocrine disruptors that addresses the needs that the U.S. EPA has already articulated in its Research Plan for Endocrine Disruptors (Research Plan; U.S. EPA 1998a) and its Multiyear Plan (MYP) for Endocrine Disruptors (U.S. EPA 2003a). It was not our purpose to create a new research agenda or to critique the existing one. In this report we summarize the findings from the review of the program by the subcommittee.

## Background

In the early 1990s scientists began to synthesize information about the potential impacts of endocrine-mediated toxicity on humans and wildlife, arriving at the hypothesis that weakly endocrine-active compounds in the environment were having significant adverse effects on public and environmental health (Colborn and Clement 1992). In response to these earlier findings, the U.S. EPA convened two international workshops in 1995 (Ankley et al. 1997; Kavlock et al. 1996) to identify uncertainties and research needs relative to future risk assessments for EDCs. These workshops reported effects on reproductive, neurologic, and immunologic function, and carcinogenesis as the major end points of concern and recommendations for research and also served as the basis for establishing national and international research efforts. The MYP (U.S. EPA 2003a) on endocrine disruptors covers a considerable fraction of the research identified by the scientific community as being important for understanding the impact of EDCs.

A recent publication identifies 10 key areas of uncertainty that must be addressed to determine the significance of endocrine disruptors as public and ecologic health threats (Daston et al. 2003). The relationship of these key research questions to the long-term goals in the MYP are shown in Appendix 2. In 1996, ORD identified EDCs as one of its top six research priorities. In the same year, through the enactment of the Food Quality Protection Act of 1996 (FQPA 1996), the U.S. Congress directed the U.S. EPA to screen pesticides for estrogenic activity in humans using validated studies or other scientifically relevant information and gave the agency discretionary authority to screen for other endocrine effects as well. The Safe Drinking Water Act Amendments of 1996 (SDWA 1996), passed in the same year, authorized the U.S. EPA to screen drinking water contaminants for similar activities. To implement the legislation, a number of scientific questions needed to be addressed and resolved through research. Consequently, the U.S. EPA EDC Research Program and the development and implementation of a mandated Endocrine Disruptor Screening Program (EDSP) by the Office of Prevention, Pesticides, and Toxic Substances (OPPTS) are on parallel yet highly interactive tracks (U.S. EPA 2005b).

The peer-reviewed blueprint for the U.S. EPA EDC Research Program was published in 1998 as the ORD Research Plan and took into consideration the advice provided to OPPTS on the implementation of the legislation by an independent expert advisory panel (U.S. EPA 1998a). Five years later, ORD developed the MYP (U.S. EPA 2003a) that identifies the elements of the Research Plan that specifically will be addressed over the next 5–10 years, intramurally, across three national laboratories and one national center and, extramurally, through a competitive grants program.

The purpose of the MYP is to provide a framework that integrates research across the laboratories and centers of the ORD to produce scientifically credible results in accordance with the Government Performance and Results Act (Office of Management and Budget 2005) goals and supports the agency’s mission to protect human health and the environment. The MYP identifies long-term goals (LTGs), and presents annual performance goals and associated annual performance measures for a planning window of approximately 5–10 years (Appendix 2). The MYP fosters the integration of strategic risk-based environmental protection and anticipation of future environmental issues by communicating the research approach and timing for responding to environmental issues. The MYP will be updated every 2 years to reflect the current state of the science, resource availability, and agency priorities, and reflects research activities implemented and planned for 2000 through 2012.

In addition to the MYP, which identifies the EDC research directions for all the laboratories and centers of the ORD, some ORD organizations have developed their own implementation plans. For example, the National Risk Management Research Laboratory (NRMRL) of the ORD has developed a Risk Management Evaluation (Sayles et al. 2002), and the National Health and Environmental Effects Research Laboratory (NHEERL) has developed a Research Implementation Plan (U.S. EPA 2004a) to guide the specific activities of that laboratory that are related to EDCs.

## Program Review Materials

The subcommittee reviewed materials sent by the U.S. EPA, including the Research Plan (U.S. EPA 1998a), the MYP (U.S. EPA 2003a), the NHEERL Research Implementation Plan (U.S. EPA 2004a), a bibliography of publications by intramural and extramural researchers, proceedings and abstracts from recent EDC workshops (U.S. EPA 2002a, 2003b), abstracts of the posters to be presented at the program review meeting, and biographical sketches of the intramural and extramural researchers. Additional reports (American Chemistry Council 1999; Damstra et al. 2002; U.S. EPA 1998b, 2002b, 2004b) were also made available to the subcommittee prior to the 13–14 December 2004 face-to-face meeting.

The U.S. EPA staff provided an overview of the EDC Research Program and the LTGs, and poster sessions were presented and discussed by intramural and extramural researchers, program and regional office scientists, and grantees. Poster sessions were followed by presentations by representatives from program and regional offices who spoke to the relevance of the research program. The meeting included an opportunity for public comment. At the conclusion of the meeting, the subcommittee presented a draft oral report of its findings.

The subcommittee organized the review based on the three LTGs presented in the MYP (U.S. EPA 2003a), commenting and responding to the first three charge questions (program design, program relevance, program progress/performance) for each long-term goal. Charge questions four and five (leadership and resource allocation) were evaluated separately, as they cross-cut the overall program (Appendix 1). The full report of the subcommittee can be accessed at the BOSC website (BOSC 2005). Highlights of the findings of the subcommittee are presented below.

## Peer-Review Subcommittee Findings

### Long-term goal 1

LTG 1 strives to provide the science underlying the effects, exposure, risk assessment, and risk management of EDCs. The goals set forth in the Research Plan and the MYP to address the underlying science needs for risk assessment and management of EDCs continue to be appropriate. The research and implementation plans to achieve these goals are well founded and provide a logical framework for attaining the Research Plan goals.

LTG 1 is well designed and takes advantage of existing core competencies in reproductive toxicology, mechanistic toxicology, ecotoxicology, risk assessment, and risk management methodology to address these questions. The capabilities of these scientists are unique in breadth, depth, and scope within the federal government. No other federal agency is equipped to provide answers regarding both risk assessment and management of EDCs. Thus, the outcomes of the Research Plan continue to provide essential, fundamental scientific support for other regulatory and resource management agencies, both federal and state, as well as external investigators and industry.

The EDC Program has relied on the Science to Achieve Results (STAR) grants program to conduct research and provide expertise to achieve program-related outcomes related to LTG 1. STAR grant recipients have contributed important findings on many topics including interspecies differences in steroid receptors, avian and invertebrate models for EDC evaluations, and the effects of multiple EDC exposures. The Research Plan has also utilized and relied on the skills and abilities of scientists from other federal agencies to complement some activities in this research area. The STAR program, therefore, is an essential element in the EDC Program to continue to meet its goals outlined in the Research Plan. One example of the plan’s reliance on extramural expertise is in avian toxicology. The expertise to conduct avian toxicology studies in the laboratory or in the field does not reside within the U.S. EPA/ORD.

The science conducted under LTG 1 is unique and provides the foundation required for future risk assessment and risk management activities legislatively mandated to the U.S. EPA. Work on development of models (mammalian, fish, amphibian, and avian) is ongoing, with appropriate end points to help identify and evaluate uncertainties for human and ecologic risk assessment of EDCs. The end points chosen are relevant for the ecologic risk assessment process. Model compounds include traditional organochlorine pesticides and industrial compounds, positive controls (estrogens and androgens), current use pesticides (e.g., atrazine), and thyroid-active agents. The chemicals chosen are timely and important relative to exposure, and low-dose effects and latent effects are being addressed in a rigorous manner. Thus, a good deal of high-quality data are being developed under LTG 1, providing the foundation for environmental risk assessments and risk management of EDCs.

The Research Plan has developed critical and relevant information on mode of action, interspecies differences, multiple chemical exposures, critical life stages, dose–response characteristics, effects at multiple levels of biological organization, linkages among assessment end points, and low-dose effects of EDCs. All of these findings are required for the appropriate evaluation and risk assessments of EDCs.

#### Strengths and challenges

The scientific expertise available in ORD and some of the external STAR recipients are a primary strength for LTG 1. The research program structure and implementation is logical and well designed, adding strengths in this key area. Additionally, the models that are being characterized to evaluate EDCs under LTG 3 will complement the on-going efforts in LTG 1.

The EDC Program and scientists within ORD provide strong leadership in the design and execution of research efforts in this area. The models that have been designed or modified to evaluate endocrine disruption will provide the data required to develop risk assessments. The models have been the subject of harmonization efforts to be compatible with Organisation for Economic Cooperation and Development (OECD) guidelines for toxicity testing.

The risk management program provides important information for the regulated community with regard to identifying and prioritizing EDCs of concern and leadership in developing risk management approaches.

The complexity of endocrine systems, in conjunction with the diversity of potentially endocrine-active chemicals makes the evaluation of combined effects of EDCs a daunting task. Even with a solid approach, good laboratory models, and adequate funding, it is likely that it will be some years for ORD to fully evaluate this question.

Scientific expertise for the areas of human and aquatic (fish, invertebrates, and amphibians) species is very good. However, this same strength is not apparent in the area of wildlife toxicology, and much of the experimental research and expertise comes in the way of STAR grant recipients. It is certainly advantageous to utilize the expertise of these scientists from outside the agency; however, more expertise in the area of wildlife toxicology within the agency will be required to fully attain the goals within this program and meet the exact needs of regulatory concern. In addition the evaluations of EDCs on wildlife within a risk assessment paradigm, including evaluation of uncertainties, would almost certainly require full-time U.S. EPA personnel. Because of the complexities in extrapolating among the many species in the environment that may be affected by endocrine disruptors, it will be important for ORD to continue to collaborate with other federal, academic, and nongovernmental organizations, and industry partners to better characterize the range of variability among species.

The model and framework for development of critical information on EDCs for risk assessment is well established and making progress. Efforts should now focus on development of risk assessment paradigms for EDCs and application of the research findings. A major challenge for risk assessment will be to settle on definitions of what constitutes an adverse effect and what constitutes a biological indicator. This challenge does not reside exclusively within ORD, but ORD research will be needed to support decisions in this area.

The development of analytical methods for detection and quantitation of EDCs is a significant challenge and was not clearly identified as an annual performance goal or annual performance measure for this research. This may slow the progress of the risk management program. In addition, studies conducted by investigators outside the U.S. EPA have reported the use of predictive tools that can be used to prioritize the focus of selective water and wastewater treatment technologies. These findings, as well as plans for future research regarding natural processes in sediments, could be integrated into this work.

### Long-term goal 2

LTG 2 seeks to determine the extent of the impact of endocrine disruptors on humans, wildlife, and the environment. The subcommittee evaluation of the Research Plan and the MYP found that the goals and science questions are appropriate and represent an understandable and solid framework for setting research priorities for endocrine disruptors. The Research Plan has stood the test of time and is appropriately reflected in the MYP for LTG 2.

The presentations and posters under LTG 2 represented primarily issues of environmental and human exposures to actual and suspected EDCs, and the spectrum of effects that might be produced from those exposures. There is obvious overlap with the other long-term goals. For example, one of the key science questions of the MYP for LTG 2 is to determine how and to what degree human and wildlife populations are exposed to EDCs (Appendix 2). This question is also relevant to LTG 1 goals such as those dealing with dose response and exposure to mixtures of EDCs. This overlap is desirable if it is understood that the success of each of the long-term goals is dependent on continued productive interactions among all the projects covered under the endocrine disruptor umbrella as well as related activities in programs in human health, computational toxicology, risk assessment, risk management, and the needs of the regional offices. To date, the U.S. EPA appears to have been successful in linking different components of the EDC Program.

In the case of environmental releases and ecologic effects, the U.S. EPA has taken two approaches: *a*) study chemicals with known EDC activity and *b*) evaluate the endocrine activity of emissions and releases from different sources followed by attempts to identify the chemicals responsible for the observed activity. Both approaches are needed, but the U.S. EPA should not lose sight of the goal of determining chemical classes of interest and the sources of EDCs.

#### Strengths and challenges

The U.S. EPA research program relevant to the science questions contained in LTG 2 has been productive, of high quality, and relevant to the mission of the U.S. EPA. Available resources have been used efficiently, and both the intramural and extramural investigators demonstrate a high degree of enthusiasm for the projects. In general, greater progress has been made on ecologic effects of EDCs compared with human health effects, although several appropriate human health projects are underway.

Many of the models required for studying fish and invertebrate effects of EDCs have been developed, modified, and/or applied. Ongoing studies have set appropriate priorities for determining sources of EDC exposures including concentrated animal feedlot operations, combustion processes, and pulp mills. The ecologic studies have effectively coupled field studies, biomarker measurements, analytical chemistry, laboratory studies with whole organisms, and hormone-responsive cells with effluents suspected of possessing hormonal activity.

U.S. EPA scientists have made good decisions on how to best use genomics, with a good balance between molecular toxicology and effects on aquatic species and experimental animals. The information generated from these studies should produce important data that address critical knowledge gaps.

Interactions between ORD and the regional offices on EDC issues are strong, effective, and frequent and provide a good model for the U.S. EPA to use in other areas. The research within LTG 2 is consistent with the overarching Research Plan developed by the U.S. EPA and other agencies in 1998, with research priorities reflecting the high-priority goals identified by that plan.

Although priorities for chemicals studied have been appropriate, the U.S. EPA should strive to continue to improve its interactions with other agencies with a strong interest in the EDCs, such as the Centers for Disease Control and Prevention (CDC), National Institute of Environmental Health Sciences (NIEHS), National Toxicology Program, National Institute for Occupational Safety and Health (NIOSH), and U.S. Food and Drug Administration (FDA). These collaborations will facilitate identifying new sources of environmental and human exposures to EDCs. Moreover, the U.S. EPA should mine data made available from the High Production Volume program (U.S. EPA 2005c) and work with the U.S. FDA to investigate the role of pharmaceuticals in the environment as a source of endocrine disruptors.

The epidemiology studies represent an important component of the EDC Program relevant to LTG 2. The U.S. EPA is encouraged to continue these studies and to use the exposure results to set priorities for future epidemiology studies so that duplicative studies are kept to a minimum. The U.S. EPA should continue to investigate the common ground between ecologic and human health, as no other agency’s mission provides this opportunity.

It will be important for the U.S. EPA to take a leadership role in the application of “omics” technologies to address many of the science questions critical for evaluating environmental and human health effects of EDCs. Although there was evidence of considerable progress in this regard, future development of this approach will require a strong commitment to a systems biology approach and computational toxicology as well as effective interactions with those generating much of the basic data.

### Long-Term Goal 3

The screening and testing program of the U.S. EPA was established to comply with the FQPA (1996) and SDWA Amendments (1996). Principles for screening and testing of chemicals for potential endocrine-disrupting activity were developed by the Endocrine Disruptor Screening and Testing Advisory Committee (EDSTAC) (U.S. EPA 1998b), a federal advisory committee convened by the U.S. EPA to provide recommendations on how to implement the endocrine-disruptor assessment aspects of FQPA and SDWA. EDSTAC recommended that the evaluation of chemicals proceed in a tiered manner: prioritization for assessment, followed by screening for putative endocrine activity, which would then be confirmed by definitive testing. EDSTAC recommended that the screening encompass effects on estrogen, androgen, and thyroid hormone function.

The recommendations of EDSTAC have served as the basis for the U.S. EPA EDSP (U.S. EPA 2005b). ORD has taken the appropriate steps through its MYP to develop tools for prioritization, has standardized and validated assays for screening, and has added sensitive end points to traditional assessments of reproductive toxicity that enable a more complete understanding of the mechanisms of EDCs.

The research on screening and testing is essential to the mission of the U.S. EPA and to the mandates given to the U.S. EPA under the FQPA (1996) and the SDWA Amendments (1996). Virtually all of the short-term goals (first several years) identified under the MYP are fully aligned with the recommendations of EDSTAC and to the efforts of the U.S. EPA to comply with the nature and timing of its FQPA/SDWA mandates. Research support and expertise from ORD have been at the forefront of developing, standardizing, and validating screens for endocrine disruptors.

The program plan with respect to LTG 3 exceeds the explicit recommendations of EDSTAC and takes advantage of improvements in the science, especially in the realm of computational biology. The U.S. EPA has recently launched a national, multilaboratory computational toxicology program that stands to contribute significantly to endocrine disruptor screening, particularly through the development of quantitative structure–activity relationship (QSAR) models.

#### Strengths and challenges

The progress on LTG 3 within the EDC Program has been excellent. Two mammalian tests have already been through a validation program administered by the OECD. These should be available for use by the EDSP very soon. Development of the other two tests recommended by EDSTAC is in progress, and publications emanating from this work indicate that the work is on track.

There has been significant progress within ORD and its scientific partners in the development and validation of several relevant bioassays important for the screening and testing requirements for LTG 3. The uterotrophic assay for estrogenic effects and the Hershberger assay for androgenic effects have been the subject of multilaboratory, multinational validation programs coordinated by OECD, with considerable guidance from ORD scientists. The pubertal male and female assays, which evaluate the attainment of puberty in rodents and are semiapical in that they assess the integrated function of a number of mechanisms of action (hormone synthesis, hormone action, endocrine axes), are still under development via ORD research programs. ORD included the completion of these as short-term goals in its MYP.

*In vitro* assays for androgen receptor (AR) and estrogen receptor (ER) binding have been developed and validated. Moreover, *in vitro* AR- and ER-dependent transactivation assays in transformed cell lines are now available, and these use both stable and transiently transfected cells. These assays, coupled with ongoing studies in other laboratories, suggest that this important screening component of LTG 3 is nearly complete. In addition, an *in vitro* assay for determining the effects of EDCs on steroidogenesis has been developed, and the approach will be capable of measuring both modulation of steroidogenic gene expression and activity. This bioassay seems highly promising and requires further validation using more extensive sets of test EDCs and possibly development of alternate cell lines to determine possible intercellular differences in response to EDCs. Excellent progress has also been made on validation of short-term *in vivo* assays for the determination of estrogenic/antiestrogenic and androgenic/antiandrogenic chemicals, using the rat as a model.

The EDC research is mechanistically driven, which provides a solid scientific foundation for the test methods that are developed. Because of this mechanistic focus, it is highly likely that the methods developed will be valid, broadly applicable, and easily interpreted.

Clear goals are articulated for the development of screening and testing methods for endocrine disruption. U.S. EPA research is well coordinated with other federal agencies and with international efforts on the standardization and validation of endocrine-screening assays. ORD has been highly responsive to the needs of the EDSP (U.S. EPA 2005b) and has provided technical expertise to the Office of Science Coordination and Policy. ORD has used its leadership role in the fields of reproductive, developmental, endocrine, and aquatic toxicology to adapt and develop methods that have high relevance to the needs of the program offices and to the protection of public and environmental health.

The major challenge that ORD has faced is handing off its research to the program offices so that validation and implementation can occur in a timely manner. Much of the delay in validation and regulatory acceptance, however, is because this process takes place largely outside the agency. The transfer of protocols to contract laboratories has been problematic. This has led to *a*) a substantial commitment by the U.S. EPA staff to refine and troubleshot assays, and *b*) a negative effect on other core research activities that are the responsibility of the U.S. EPA staff. The subcommittee recommends that there be a mechanism in place to ensure the timely transition of protocols to the OPPTS.

Research within NHEERL has contributed to basic understanding of the toxic responses to estrogens, antiandrogens (within the Reproductive Toxicology Division) and thyroid toxicants (within the Experimental Toxicology Division), which in turn has led directly to the development of improved methods for EDC detection. This research is diffuse and is occurring in multiple divisions within NHEERL; many of the accomplishments in these areas have been difficult to capture in the list of annual performance goals. The subcommittee recommended that the U.S. EPA try to summarize this research and its relevance to EDC identification in subsequent reports and revisions of the MYP.

ORD is beginning to develop core competencies in genomics and QSAR methods, both of which hold promise in EDC identification. Because these areas are so data intensive, it will be important for ORD to train or hire experts in bioinformatics to work with the life sciences experts already on staff.

## Program Leadership and Resources

The EDC Program has enjoyed outstanding leadership since its inception in 1995. The EDC Program scientists consult with and provide technical assistance to other U.S. EPA program offices, other federal agencies, and within the broader scientific community. EDC scientists are engaged in intramural and extramural research within ORD, and program scientists have provided exemplary leadership in the field at the national and international level. The EDC scientists are at the forefront of research in this field in EDC screening and testing methodologies for mammalian and ecologic tests, source identification, effects on wildlife, and ecologic health.

The EDC Program is unique in that no other U.S. federal agency has an EDC program with such broad responsibilities. The EDC Program is not just an umbrella for a series of independent project but a fully integrated program across all the laboratories and centers (with the exception of the Homeland Security Research Center). The program is nationally and internationally recognized as a multidisciplinary set of research projects for both human health and wildlife and cuts across the risk assessment/risk management paradigm.

The EDC Program was projected to have an average annual budget of $12 million. This figure includes the STAR grants program, which averages $4 million in years when it is funded. In actuality, the average annual budget from fiscal year (FY) 2003–2005 has ranged from $12.7 million enacted in 2003 to the FY 2005 request of $8.0 million, which includes approximately 55 full-time equivalent personnel per year. The EDC Program director does not have direct access to human or financial resources to carry out the objectives of the program. Instead, the director must negotiate with the division heads of the laboratories and centers of ORD to use the time and effort of scientists with the needed expertise.

The laboratories and center that contribute resources to the EDC Program are NRMRL, National Center for Environmental Research (NCER), NERL, and NHEERL. Although the total budget for the program has decreased since FY 2003 (from $12.7 million to an $8.0 million request for FY 2005), the percentage of resources provided by each of these laboratories has been relatively stable (except for NCER) over the past 2 years and is in proportion to the number and extent of tasks that they perform for the EDC Program across the long-term goals. The STAR grants program adds significant value to the research portfolio of the EDC Program. The research sponsored by the STAR program assists in filling identified research gaps, brings in research expertise that is not found among intramural scientists, and assists the ORD in responding to new issues that the laboratories and centers may not be able to readily address.

The manner in which this program is funded, though indirect and possibly cumbersome, does not appear to hinder the quality of the research being done in the program. It is apparent that the EDC Program director has had success in convincing division directors to loan scientist time to the EDC Program. It does make it more difficult, however, for the program director to do forward planning or to plan to investigate emerging issues. ORD has been very astute in leveraging the resources of the EDC Program by collaborating with other federal agencies. The amount of research done by the EDC Program has been expanded by collaboration with agencies such as NIOSH, NIEHS, and the National Cancer Institute; however, the fragmentation of scientists’ time without compensation raises concern about whether the productivity (number of manuscripts published, etc.) of these scientists is negatively impacted by participation in the EDC Program.

The situations cited above (insufficient funding and the mechanism used to provide resources to the EDC Program) can be remedied by several courses of action: *a*) hiring additional personnel to share the workload of the participating laboratories; *b*) elevating the position of the EDC Program director to the level of the laboratory/center directors; and *c*) giving the EDC Program director budget authority. These actions would allow the program director to negotiate for needed research expertise from a position of strength and allow the program director to enhance the laboratories that participate in EDC Program research. The ORD has plans to enact the latter two of these ideas in the near future for all newly hired national program directors.

## Conclusions

The goals and science questions are appropriate and represent an understandable and solid framework for setting research priorities for EDCs. The Research Plan was formalized in 1998 after a series of workshops, interagency considerations, and meetings that embraced all relevant stakeholders. The program is nationally and internationally recognized as a multidisciplinary set of research areas for both human health and wildlife and cuts across the risk assessment/risk management paradigm. Key research areas are closely aligned to the LTGs and annual performance goals. The EDC Program is a combination of problem-driven and core research that has stood the test of time.

The subcommittee is favorably impressed with the quality and relevance of the work and the progress to date, although we recognize that much remains to be done. The annual performance goals are highly ambitious and it should be recognized that progress on those goals will, in most cases, continue well past the initial timelines. We are impressed with the enthusiasm of the investigators and their commitment to addressing the difficult and controversial issues that surround endocrine-disruptor research.

One of the main functions of the U.S. EPA is risk assessment and risk management of chemicals in commerce and the environment. LTG 1 provides a solid scientific foundation for conducting risk assessments and making risk management decisions related to endocrine disruptors. The research that falls under this goal covers the major questions in the key areas of the risk paradigm. Although there are many challenges to fully defining the nature of possible biological effects and the extent of exposure, the research being carried out under this long-term goal will put the U.S. EPA in a strong position to make scientifically grounded decisions.

The research program relevant to the science questions contained in LTG 2 has been productive, of high quality, and relevant to the mission of the U.S. EPA. Available resources have been used efficiently, resulting in a high degree of enthusiasm for the projects by both the intramural and extramural investigators. In general, greater progress has been made on ecologic effects of EDCs compared with human health effects, although several appropriate human health projects are underway.

The progress on LTG 3 within the EDC Program has been excellent. Two mammalian tests have already been through a validation program administered by OECD. These should be available for use by the EDSP very soon. Development of the other two tests recommended by EDSTAC is in progress, and publications emanating from this work indicate that the work is progressing appropriately on track. ORD has articulated clear goals for the development of screening and testing methods for endocrine disruption and is fulfilling those goals in an admirable fashion. The research is directly relevant to legislation that the U.S. EPA administers and is serving the program offices well. Research of the U.S. EPA is well coordinated with other federal agencies and with international efforts on the standardization and validation of endocrine-screening assays.

Despite good progress, the future success of the U.S. EPA in meeting the specified goals of the EDC Program will depend on a number of factors, including *a*) strengthening their expertise in wildlife toxicology; *b*) expediting validation of the EDSTAC tests; and *c*) taking a leadership role in the application of omics technologies to address many of the science questions critical for evaluating environmental and human health effects of EDCs. In addition, support from U.S. EPA management, multidisciplinary intramural research spanning ORD and other U.S. EPA entities, extramural grants, and continued interagency collaborations (e.g., NIEHS, CDC, U.S. Geological Survey, U.S. FDA, U.S. Department of Agriculture, and others), especially with regard to identifying new sources of environmental and human exposure, will be critical. To date, the EDC Program has been very astute in leveraging its resources by collaborating with other federal agencies and has extended its research program as a result of these collaborations. The continuation of the external grants program, in particular the STAR grants program, is vital, as it provides a mechanism for the U.S. EPA to more efficiently evaluate new technologies and innovations for use in the risk assessment and risk management arenas.

## Appendix 1. Charge questions for Endocrine Disrupting Chemicals Research Program review

### Charge Question 1. Program design

- Do the goals and priorities of the Endocrine Disruptors Research Plan (Research Plan) and MYP, including the MYP’s long-term goals (LTGs) of the MYP, represent appropriate outcome measures for this program?
- Has the research program appropriately implemented the Research Plan and the Office of Research and Development (ORD) MYP, tracking the key science questions closely and describing clearly the expectations for providing answers to the key science questions?
- Do the Research Plan and MYP of the ORD make it clear what the unique research niche of the ORD is in the context of endocrine disruptors research being conducted across the federal government and internationally? Is the rationale sound for supporting the choices that ORD has made in the past and for the future regarding what to emphasize over the next 5–7 years? If not, what arguments need to be more clearly stated, and what additional evidence and information need to be included?
- Have the potential public benefits of the Research Plan been clearly articulated? Are there interagency collaborations that should and can be improved to advance the research agenda of the agency? Too what extent has the U.S. EPA established and utilized other agencies (inside and outside the government) in advancing the research agenda of the U.S. EPA? What are the impediments to collaboration with other organizations?
- Are the research products (annual performance measures) and their sequencing and emphases over the next approximately 5–7 years appropriate, especially in light of needs for the Endocrine Disruptor Screening Program by the Office of Prevention, Pesticides and Toxic Substances? Does the program have a complete schedule with annual milestones for decisions and termination points, highlighting changes from previous schedules?
- Is the MYP sufficiently flexible to adapt to anticipated future science and policy direction changes?

### Charge Question 2. Program relevance

- To what extent has the research program, as evidenced by the Research Plan, the ORD MYP, National Health Effects and and Research Laboratory Implementation Plan, and other submitted documentation been responsive to agency and other stakeholder needs and priorities?
- What role have program scientists had in providing technical support to agency program and regional offices?

### Charge Question 3. Program progress in addressing key scientific questions and impacting environmental decision making

- What degree of progress has been made in addressing each of the LTGs and associated key research questions?
- To what degree are scientific products being used in environmental decision making?
- Has the Research Plan met its annual performance goals?

### Charge Question 4. Program contributions to scientific leadership

- To what extent have the program and its scientists contributed to advancing the state of science on endocrine disruptors?

### Charge Question 5. Program resource allocation

- The MYP was developed based on an assumption of level resources (approximately $12 million including approximately 55 full-time equivalent personnel) over the period covered by the plan.
- Is the relative allocation of resources across the LTGs adequate based on consideration of scientific and programmatic needs?
- Is the manner in which resources are allocated appropriate?
- Do these funding processes maintain program quality?

## Appendix 2. Key research questions and relationship to long-term goals in the U.S. EPA EDC Multi-Year Plan

### Long-Term Goal 1. Provide a better understanding of science underlying the effects, exposure, assessment, and management of endocrine disruptors

- What approaches are needed to assess risks to humans and wildlife?
- What are the dose–response characteristics in the low-dose region?
- What extrapolation tools are needed?
- What are the effects of exposure to multiple EDCs, and will a toxic equivalency factor approach be feasible?
- What is the nature and manifestation of latent effects from developmental exposures to EDCs?
- How can unreasonable risks be managed?

### Long-Term Goal 2. Determine the extent of the impact of endocrine disruptors on humans, wildlife, and the environment

- What effects are occurring in exposed humans and wildlife populations?
- What are the chemical classes of interest and their potencies?
- How and to what degree are human and wildlife populations exposed to EDCs?
- What are the major sources and environmental fates of EDCs?

### Long-term Goal 3. Support the screening and testing program of the U.S. EPA

- Do our testing guidelines adequately evaluate potential endocrine-mediated effects?
